# Obstetrical Society of Philadelphia

**Published:** 1889-12

**Authors:** J. M. Baldy

**Affiliations:** Secretary; 328 South Seventeenth Street


### OBSTETRICAL SOCIETY OF PHILADELPHIA.

THURSDAY, OCT. 3, 1889.

DR. THEOPHILUS PARVIN in the chair.

Dr. E. P. BERNARDY.—Suppurating Post-Puerperal Hæmatocele. Operation. Cure.

Mrs. McM., age thirty-eight, sixth pregnancy. Fell in labor April 19, 1889, about eight o'clock in the evening; head presentation, right occipito-posterior position; delivered about midnight of same evening of a large living male child. Labor seemed normal, but somewhat tedious in regard to her former labors, no doubt caused by the position of the head, which gradually rotated to an anterior occipito. There was a constant aching feeling between the pains, not that absence of pain generally expected in natural labor pains; as the patient remarked, it felt like a bad toothache. Third stage normal; placenta came away within twenty minutes; uterus contracted rapidly and firmly. Shortly after the termination of labor, the muscles of the calf of the left leg became painfully cramped, lasting nearly an hour; hot applications and rubbing seem to relieve the patient. Pulse about eighty, but weak.

About six o'clock in the morning, the husband called at my office and stated that about four o'clock his wife had been taken with a sudden sharp pain under the right breast, which prevented her breathing freely. Ordered teaspoonful of liq. morphiæ sulph. every half hour, and warm applications to the side. Saw her shortly afterward; pulse 120, temperature 101°. Sharp agonizing pain below right breast; impossible to take a full breath. Auscultation negative; no pain over the abdomen, strong pressure being made over its entire surface.

April 23, pain under breast relieved, sense of fullness of the abdomen, constipation, cannot pass her urine; urine drawn off by catheter; one drachm Rochelle salt every hour until free purgation took place. Next day pulse quick and irritable, temperature 103°, tongue dry, still unable to pass her urine, bowels open freely; complains of a sense of fulness in the rectum. Examination by the rectum shows a tumor about the size of an egg pressing in its walls; vaginal examination reveals a tumor behind and to the left of the uterus. Ordered hot vaginal injections of $\frac{1}{1000}$ bin. iod. mercury, poultices over abdomen; there being no positive pain, only a feeling of fullness, no opium was given. From this time up to the day of operation (May 5, 1889) the temperature varied from 102° to 104°; under large doses of sulphate of quinine it would drop.

April 27, 1889, examination by rectum and vagina shows the tumor increasing rapidly; it is now the size of a large orange, and very sensitive to the touch.

May 1, the tumor now fills the entire rectal cavity, pushing down to almost the external sphincter. The entire left side of the pelvis is completely filled by the tumor, pushing the enlarged uterus, which is movable, well to the right side, making it appear as if there were another tumor. Advised operation.

May 3, case seen by Dr. Joseph Price; same condition as on May 1. Patient hectic toward the afternoon; face has assumed a pecular yellowish appearance; eyeballs yellow.

May 5, operation. Present, Drs. Joseph Price, M. Price, Solis-Cohen. Operator, Dr. Eugene P. Bernardy. The usual medium abdominal incision was made; on introduction of the finger in the abdomen, the entire left side of the pelvis behind the uterus and broad ligament was found filled by a tumor, surrounded by adhesions; slight adhesions to the intestines, which were readily separated before enucleating the left side tumor. In passing the finger behind the right side of the uterus, the finger ruptured some slight adhesions, entering a cavity from which freely flowed thick black blood, looking and smelling like blood contained in an extra-uterine sac. After enucleating the left side tumor, it left an immense cavity, which was, as well as the abdominal cavity, well douched with hot water; both ovaries and tubes were healthy, and were not touched; glass drainage-tube introduced, and abdomen closed.

On the day of the operation, the pulse was 120, temperature 102°. The day following, pulse 96, temperature 101°. Third day of operation, temperature and pulse normal. On the sixth day, glass drainage-tube changed to rubber tube. Three days later, tube taken out; stitches taken out the sixth day; union except where drainage is placed. May 23, 1889, entire cut healed, and patient discharged during the first week in June.

DR. J. PRICE.—I saw this patient with Dr. Bernardy and Dr. Cohen; and to have a purely medical man urge the importance of abdominal section in a post-puerperal case is very encouraging. There was a considerable quantity of broken-down blood, which was washed out with difficulty. If the case had not been a post-puerperal one, the history would have been that of an extra-uterine fœtation. This case demonstrates most forcibly the fallacy of claims made in regard to refinements in diagnosis, and shows the folly of claiming a positive diagnosis.

Dr. H. H. KYNETT.—I report the first two cases because they present important features for consideration in the treatment of pelvic troubles. The specimens of the first case I am not able to show to-night, owing to the lapse of time since removal.

CASE I.—L. P., colored, age thirty, married seven years, and never pregnant; complaining more or less ever since marriage. Abdominal section April 2, 1889. Removal of both appendages for double pyosalpinx and double ovarian abscesses; release of adhesions, irrigation and drainage. When the peritoneal cavity was opened, there was a free discharge of muddy, blood-stained fluid, indicating a marked peritonitis. Investigation showed this fluid was contained in a sac formed by inflammatory processes, shutting off the pelvic portion from the general peritoneal cavity. The adhesions, however, were friable and easily broken. Contained in this pelvic abscess cavity were four distinct pus-sacs; viz., two huge pus-tubes and two ovarian abscesses; the larger the size of an orange.

The removal of these sacs was not difficult; the patient recovered promptly, and is now in better health and spirits than since marriage.

Points worthy of notice in this case are:

1st. Four distinct abscess cavities within a fifth. Query, what would have been the result of Martin's treatment of pelvic abscess by vaginal drainage?

2d. In spite of careful manipulation, both ovarian abscesses were ruptured in removal, and the walls of the containing sac were very easily broken up. Query, what might electricity, properly applied, have accomplished?

3d. When first seen, the patient did not complain of symptoms of acute trouble at all commensurate with the condition revealed.

4th. Menstruation has occurred regularly since the operation, bleeding being profuse and lasting three days.

5th. Both patient and her husband gave unquestionable histories of gonorrhœa.

The second case was operated on to produce premature menopause for a rapidly growing fibroid uterus.

CASE II.—L. A., white, age twenty-eight, married six years, no children. Patient believed that she miscarried twice when about two months pregnant, the last time four years ago. Since puberty, menstruation has been very profuse and painful. For the last four years she has been bleeding three-quarters of the time, and latterly has been incapacitated for work. Abdominal section September 11, 1889. Removal of both appendages for double hydrosalpinx and left-ovarian cyst. There was also a small cyst in the right ovary. The adhesions were universal and exceeding tough, making the removal difficult. The uterus was large and hard; irrigation and drainage.

Patient made an uninterrupted recovery, and is now sitting up. This case is particularly interesting, as before operation it seemed a fit case for electricity. The uterus being high in the pelvis and large, the condition of the appendages was not easily discoverable. Irrigation and drainage were used for fear of hemorrhage from the separated adhesions.

The third case is interesting on account of the comparative variety of the tumor.

It was removed September 8, 1889, from the breast of Mrs. P., white, age thirty-two, married six years, two children. When the patient began to menstruate at the age of sixteen years, she first noticed a small lump in the inner of left breast, on a level with the nipple. It occasioned no trouble. It remained quiescent during her first gestation and nursing; in fact, until three months after her second child was born, when it began to enlarge. She never had any difficulty in nursing, but remarked that after the tumor began to grow she had less milk in the left breast. At this time, also, the tumor pained her for a few days, and led her to fear an abscess. The pain subsided, but the enlargement continued. She was afraid of cancer, and desired the tumor removed.

When seen, the tumor was somewhat larger than it now is. The skin was normal in appearance and freely movable over the mass. The superficial veins were enlarged. The nipple was not affected, and the growth appeared outside the areola. It was firmly adherent in the glandular structure of the breast, and required dissection by the knife. Its contents had the greasy, sticky, cheesy appearance of a dermoid cyst. There were no other points of thickening or hardness discovered in the breast.

I believe it to be a solid milk cyst. Microscopic examination has not yet been made.

REMOVAL OF A LARGE OVARIAN CYST, FOLLOWED BY RUPTURE OF THE RIGHT COMMON ILIAC VEIN.—BY W. L. TAYLOR.

The patient, of whose condition I beg to present the following history, was sent to me by Dr. D. L. Hetrick, of Bedford county, after he had diagnosed the existence of an ovarian cyst.

Miss L. M., age twenty-four, single, tall, very much emaciated; abdomen enormously extended; puberty at 19; menses regular for three years, until July, 1887. Two weeks before her menstrual period, whilst in the harvest-field, after drinking a large quantity of cold water, had a severe chill. Menses failed to appear in July and August. In September, 1887, the menstrual flow reappeared, but there was no discharge again until March, 1888, when there was a slight flow for three or four periods, disappearing then until after the operation. In November, 1887, had an attack of malarial fever, but never was well after the chill in July. After this attack of malaria, a lump appeared in the right side of abdomen, which never caused any pain, but only a sense of discomfort from pressure, and which increased rapidly in size.

Upon examination, the abdomen gave evidence of the presence of a very large encysted fluid, ovarian in character. On July 7, I operated with the assistance of Drs. W. A. Carey and E. R. Kirby, and removed a non-adherent cyst of the right ovary. The fluid of the cyst was syrupy and very heavy, weighing fully fifty pounds. The pedicle was unusually thick, and was tied in sections, and finally with a Tait ligature. The steps of the operation were devoid of special interest, and but little cyst fluid or blood escaped into the abdominal cavity. This was thoroughly washed out, and I remarked the absence of bleeding-points, and proceeded to protect the intestines preparatory to the insertion of my parietal stitches. Noticing a slight oozing of blood from the region of the pedicle, I investigated, and found that a couple of veins, which were greatly distended, had ruptured just beneath my ligatures. These I tied securely, and removed clots. Whilst doing this, I noticed higher up—fully as high as the sacro-iliac juncture, and to the right side—what appeared like an adherent intestine, rapidly distending, with a central portion most distended. This rapidly thinned out, and gave every appearance of speedy rupture. Touching it gently with my finger, it burst instantly, and there was a frightful gush of blood. I quickly grasped with my fingers the bleeding vein, for such it proved to be, and once more it broke down. I then caught it with a large Pean forceps, which imperfectly controlled the hemorrhage, and, guiding with my left index finger a large curved needle, I separated the vein from its artery and carried ligatures securely around it. These immediately stopped all hemorrhage, but caused a very decided and alarming venous swelling on either side of my ligatures. I removed the large quantity of blood carefully with my hands, and, fearing to even irrigate, closed up after introducing a drainage-tube. At the close of the operation, which was lengthened by the hemorrhage from three-quarters of an hour to nearly two hours, the patient's pulse was 160, temperature subnormal, and respiration about 40. Everything certainly pointed to a positive recurrence of hemorrhage, and she was most carefully watched.

The amount of bleeding, as shown by the drainage-tube, for the first twenty-four hours was small, comparatively speaking, one to one and a half drachms of bloody serum being removed about every two hours. On the third day the bleeding was more profuse; as much as one-half to three-fourths of an ounce was drawn off several times. But this rapidly diminished in quantity and the tube was removed on the sixth day. On the fourth day the temperature rose to 102°, but quickly dropped to from 99° to 101°, the pulse remaining very frequent, about 120, until far along in convalescence. There never was at any time any evidence of interference with return circulation in right leg. There was, on about the fourth or fifth day, slight pain in right leg; but this also occurred in the left leg, and was rheumatic in character. Convalescence was rapid and uninterrupted, and the patient returned home in about four weeks. The size of the vein from which the greatest hemorrhage occurred was, without doubt, much increased at the point of hemorrhage. This dilatation fitted in a sulcus in the cyst wall, and needed only the removal of its support, —the cyst wall—and the sudden reflux of blood, to cause its over-distention and rupture. Its location and relation to the artery and size proved it to be the right common iliac. The possibility of such a varicosed condition of either of the iliac veins should deter us from emptying a large cyst too quickly, or from turning it out whilst but partially emptied. A smaller canula and complete removal of fluid before the sac is drawn out would be much safer, and render less likely an accident which, though infrequent, is yet possible. Here and there, filling up the sulci in the tumor wall, or the interstices between lobules, these large veins are apt to distend, and the greater the pressure on either side, the greater will be this distention and thinning of the coats of the vein to the extent of the space. As long as the return of blood is hindered by the pressure of the tumor, and as long as this thin-walled venous sac has the support of the tumor wall, there is but little risk of rupture from over-distention. But remove this support suddenly, remove at the same time this interference with circulation, and we have, as in my case, a hemorrhage almost uncontrollable. It is almost impossible to conceive of ligation of such a large and important vein without some interference with circulation, at least some œdema. But collateral circulatien is plentiful between the two sides, and in my case all the veins were so enormously distended below the tumor that a compensatory circulation was soon established.

Three months after operation, Dr. Hetrick kindly writes, stating that our patient is rapidly gaining flesh and is well.

Dr. WILLIAM GOODELL.—This seems to be an unique case. I have never met with anything of the kind. The theory of varicose condition of the veins is a plausible one. I have never seen anything like it in simple unadherent cysts. In intra-ligamentary cysts I have often torn deep-seated veins, and have had difficulty in checking the hemorrhage.

Dr. DRYSDALE.—Accidents of this kind must be very rare. I have never met with anything of the kind. I imagine that it could only happen where the walls of the veins are diseased, or torn during the operation.

Dr. E. W. CUSHING, BOSTON.—I have no knowledge of any case of rupture of a vein during operation, except from injury. I do not see how the removal of pressure could cause rupture in one place, where all of the veins are varicose, although I have known this to cause syncope.

Dr. J. PRICE.—I think there is great danger of wounding the vein by the use of the Baker Brown or Peaslee needle. I think that there is one case on record in which the operator stuffed towels into the abdomen, and put the patient in bed to die, without any attempt to secure the offending vessel. These accidents have occurred from traumatism, from manipulation and wounds made by the use of instruments.

Dr. WILLIAM L. TAYLOR.—The hemorrhage occurred so long after any traumatism could have happened, and was so much higher than the pedicle, that I think it cannot be attributed to traumatism. The hemorrhage was spontaneous. It did not occur gradually, but there was a sudden gush of blood following the touch of my finger.

REPORT OF A CASE OF TUBAL PREGNANCY, WITH SPECIMENS.—PROBABLE DIAGNOSIS, AND REMOVAL PRIOR TO RUPTURE.—BY THEOPHILUS PARVIN, M. D.

Mrs. Mary E. W——— was brought to the hospital of Jefferson Medical College, September 19, suffering from a probable ectopic gestation. She is twenty-six years old, been married seven years, and has had three living children, all of whom she has nursed, and during each lactation menstruation has regularly occurred; her youngest child is thirteen months old, and she is now nursing it. About the 29th of June the usual flow began, but has continued ever since, with brief intermissions, under the ι se of medicines given by her physician, Dr. Horrowitz. In the latter part of August and in September she has suffered somewhat from nausea; and this, with some other things, would have led her to believe she was pregnant, had not the hemorrhage from the uterus been so constant. About the 1st of September she began to suffer with occasional violent attacks of pain low down in the left side, followed by much soreness; these attacks were especially liable to occur when she lifted her child.

Because of the temporary absence of Dr. Horrowitz from the city, she went some three or four times to the dispensary of the Pennsylvania Hospital, where she was examined by Dr. Baldy and Bradford, who found a tumor on the left side of the uterus, tender and cystic, with a history of almost continuous bleeding for some weeks, there being, however, no signs of any shreds; pain in the lower part of the abdomen; only very meagre symptoms of pregnancy. A probable ectopic gestation was diagnosed, and operation urged.

The day that she came to Jefferson Hospital, she had with the hemorrhage a discharge of small, membranous fragments; whether decidual or not, of course, could only be certainly known by microscopic examination. A day or two before some similar fragments were discharged, according to her statement.

Upon examination, I found a tumor adjacent to the uterus upon the left side, the uterus somewhat enlarged, and very great sensitiveness to pressure, both in the vagina and the lower part of the abdomen, especially at the left side.

The history, the examination, and the previous examinations of Dr. Baldy, with his conclusion, give me little doubt that the case was one of tubal pregnancy.

Abdominal section was done on the 20th of September, Dr. Baldy kindly being present, and Dr. W. E. Ashton assisting in the operation. The gestation cyst included in the tube was removed, and the specimen is now shown you.

The patient's convalescence has thus far been uninterrupted, almost two weeks having elapsed since the operation.

### REPORT OF A CASE OF TUBAL PREGNANCY, WITH SPECIMEN.—NON-DIAGNOSIS, BUT REMOVAL PRIOR TO RUPTURE. RECOVERY.—BY J. M. BALDY, M. D.

Mrs. G. R——— (colored) walked into the out-patient clinic of the Howard Hospital, July 5th, 1889, suffering from pain in her abdomen, so similar to that which I have often seen go with a pyosalpinx, that I diagnosed this disease before examining her. The examination revealed a large, apparently tortuous, tender mass, posterior and slightly to the left side, giving a boggy feel to the touch. The diagnosis was verified, a saline purge given, and an immediate operation advised.

One week later, a messenger summoned me to the home of the patient, where I found her lying on the bed, suffering from severe pain in the abdomen. She stated that the night before a fit of sneezing had given her a similar pain. She arose from the bed and walked to the table, a distance of about six feet. While standing there she was seized with a severe cramp-like pain, which doubled her up, and lasted for a few moments. The idea took possession of me that she possibly was suffering from an ectopic gestation. I helped her on to the bed, and made a most careful examination, which revealed the following: she had been having children at regular intervals of about two years. She had a child just a year and a half before, since which time her menstruation had been pretty regular. With the exception of an occasional "pain in her belly," she had always been well. Before coming to me she had been bleeding irregularly for about two weeks. The show was and had been clear blood, without any signs of shreds. Her husband had been away from home for about three weeks. The pain in the abdomen had been there since the bleeding began. There was not the slightest sign of pregnancy, either subjective or objective, nor did she think she was pregnant. A re-examination of the pelvis showed only what had been before found; viz., a cystic mass, which did not pulsate, posterior and to the left; apparently a distended tube. The uterus was in position of normal anteflexion, and there was a perfectly normal cervix for a multipara, There was an elevation of temperature, and the woman had had chills and creeps. I asked Dr. Hamill to see the patient for me, and he verified my examination throughout. Together we decided that it was a case of pyosalpinx, stating, at the same time, that we had thought of ectopic gestation as a possibility, but were wholly unable to find sufficient data on which to verify our suspicion.

On July 13th I opened the abdomen, with the assistance of Drs. Hamill and Naylor, and in the presence of three or four other gentlemen, and removed a left tubal pregnancy, which I here present to you. As I tore away the adhesions which bound the mass to the pelvic walls, the cyst was ruptured and a teaspoonful of black clots was discharged from the sac itself. It was evidently a pregnancy of the fimbriated end of the tube, which had become adherent to the pelvic wall, the pelvic wall thus forming one side of the sac. The case well illustrates the difficulty, nay impossibility, of at times diagnosing ectopic gestation. In three weeks the patient was sent home, and is to-day in her usual good health.

I would emphasize the following points, viz.:

The case is one of primary or unruptured tubal pregnancy. (There are now four such cases on record from this city alone; viz., Dr. J. Price's, Dr. Goodell's, Dr. Parvin's and my own.)

The patient is a colored woman, which is rather rare.

The patient did not have a long period of sterility, but was bearing children regularly.

There was at no time a sign of a decidual discharge.

There was at no time the slightest subjective or objective sign of pregnancy.

Dr. E. W. CUSHING, BOSTON.—The subject of extra-uterine pregnancy is one of great interest to me, and I can say, from sad experience, that it is not easy to make a diagnosis. After some obscure symptoms of irregularity of menstruation, etc., a near relative was taken suddenly with a severe attack, which, after the event, I felt was due to a tubal pregnancy ruptured into the broad ligament ; she finally recovered without operation. This turned my attention to the subject, and I looked up the specimens in the Harvard Medical School, which Dr. Parker photographed and I published. In another case, of which I saw the specimen, a gentleman operated for an ill-defined tumor. The cyst was opened after the operation, and a fœtus three-fourths of an inch in length found. There had not been a suspicion of pregnancy. I believe that almost every one agrees in regard to the difficulties of diagnosis, and I believe that pretty much every one here agrees as to the necessity for surgical treatment ; yet as a subject for debate here, I would suggest, in opening this discussion, that there may be cases where a man may suspect extra-uterine pregnancy, but yet be not sufficiently certain to operate, or not be able to get permission to do so; or he may be unable to do an abdominal operation himself, or secure the services of one that can. I would suggest that under such circumstances the use of the faradic current is not only justifiable but prudent. This would be proper only in the earliest stages, before the fœtus has reached such development that it would leave behind a source of irritation and suppuration. I think that the condemnation of the electric treatment in the early stage has been too sweeping and severe. Certainly the horrible cases which are recorded from attempting to puncture the fœtal sac, especially at a later date, are not likely to be repeated.

Dr. WILLIAM GOODELL.—In regard to the electrical treatment of extra-uterine fœtation, I must confess that I was theoretically inclined to believe in it. But when I had met with cases of extra-uterine fœtation, and I saw the mass that was present, and the adhesions and injuries which adjacent organs had sustained, I could no longer uphold it. In my opinion electricity should be reserved for those cases in which the woman absolutely refuses any surgical operation, or when the physician is not a laparotomist, and he cannot secure the services of one. The amount of adhesions is, however, so great, and the injury done the appendages so severe, that the woman cannot in any case conceive on that side. This was apparent in the case reported by me to the society, in which I operated previous to the rupture. In this case, indeed, the appendages of the unimplicated side were so diseased as to need removal. The operation is therefore warranted, if, for no other reason, simply for the diseased tubes and ovaries. I have practically been converted to the belief that electricity, and particularly electrolysis, should not be used in these cases. The electrolytic action is a most dangerous one. Although advocated by Apostoli, the results have been most disastrous in the cases in which it has been tried.

Fifteen years ago, in a case which I now believe was of extra-uterine pregnancy, I punctured the tumor with an aspirating needle. In a few days septicæmia set in, and the woman died.

The injection of morphia as recommended by Winckle and by others has met with better success. But while it destroys fœtal life, it cannot cure the injuries sustained by the appendages for which the best remedy is the knife.

I have had four cases of early extra-uterine pregnancy within a few months, all of which were successful. One I supposed to be a simple case of disease of the appendages, and operated accordingly. It had burst, and without marked symptoms. In another case of ruptured sac, the specimen was perfectly analogous to that presented by Dr. Baldy, and he was present at the operation. The history of the third case I have already given to the Society, and I presented the specimen. I diagnosticated the ectopic pregnancy and operated before rupture. The fourth case was one of interstitial pregnancy, on which I operated the day before I went to Europe. The woman was brought to me by her physician, with the history of hemorrhages and great suffering, but with none of pregnancy. Her physician thought that the tumor was either a fibroid or a polypus. I found a fluid tumor bulging into the endometrium, and slightly dilating the os uteri. My diagnosis was a necrotic intra-mural fibroid tumor. Using Adam's subcutaneous saw, I cut into the mass, and removed a quantity of grumous blood and broken-down fragments. These latter were examined under the microscope and found to be placental tissue. The subject of extra-uterine fœtation is of great interest, not only before and after rupture, but also in relation to the cases that go on to term. I shall never forget the first case of extra-uterine fœtation that I saw. It was the classical case of the late Dr. Parry,—the one which led him to write his admirable essay on the subject.

A distinguished surgeon was called in. This was in the days when we made marked distinction between pelvic cellulitis and pelvic peritonitis. He diagnosticated the case as one of pelvic cellulitis. There was heat and great pain, with complete immobility of the womb. The patient did not improve, and I was called in, and diagnosticated the case as one of pelvic peritonitis, which in one sense it was, as the subsequent history will reveal. My treatment was not satisfactory, and I lost sight of the case. Several weeks afterward, while I was confined to my house for a few days by an illness, Dr. Parry came to see me. He sat down by my side, and after asking about my health, he referred to this case, and pointing his long forefinger at me said: "You have made a great blunder." He then told me that he had been called in the day before to see the case, and that he considered the case one of normal pregnancy, for he had heard the heart sounds with the utmost ease; in fact, had never heard them so distinctly before. I said to him: "Dr. Parry, of course you are right in the theory of pregnancy; but depend upon it, there is something wrong, for I can hardly think that either Dr.——— or myself could have made such a mistake without some good reason for it." The time for labor came, but it did not set in. Then the death of the child occurred. Dr. Parry was now again sent for, but this time he was unable to find the os uteri, so he sent for me. With great difficulty I found the cervix above the pubic bone. We then made the diagnosis of retroverted gravid uterus. At the next visit, it intuitively flashed across my mind that it was a case of extra-uterine fœtation, and then the case was clear enough. To clinch the diagnosis, Dr. Parry introduced a hypodermic syringe needle and drew off some amniotic fluid. In a few days the head bulged down into the vagina, and we could distinguish even the sutures. We wished to incise the vagina and deliver the child with forceps; but the woman refused an operation, and died. At an autopsy, Dr. Parry withdrew the sac and fœtus and exhibited them to this Society. Not long after this I saw a second case. The woman had passed full term, the child had died, and yet labor did not come on. Her physician, much puzzled, called me in. In this case the cervical canal was open, and suspecting extra-uterine pregnancy, I passed my finger into the uterine cavity and found it empty. The case was seen by one or two other physicians in consultation with us. We were anxious to operate, but the husband would not permit it, unless we could assure him positively that his wife would recover. While waiting the woman suddenly died.

The third case I saw a number of years ago in a mulatto. The child was living at the time that I operated. There was no difficulty, either in the diagnosis or in the operation. The child, being hardly viable, gasped a few times and died. I did not dare to remove the placenta, and as the umbilical cord was very large, I made the mistake of leaving it in the lower angle of the wound as a drainage-tube. The woman died a few days later, and at the autopsy we found the liver and lungs riddled with pyæmic abscesses.

I operated on another case in which the fœtus must have died at the age of six months. The woman was perishing from blood-poisoning. She was emaciated, had high temperature and night-sweats. Pus was evidently present somewhere, and I diagnosticated the tumor as a suppurating ovarian cyst. What perplexed me was great resonance in front. At the operation this was explained by the presence of the gases of decomposition. Excessively fœtid pus escaped. I removed the fœtus, the bones of which, with the scalp and umbilical cord, were the only parts intact. The placenta was not to be found. The woman died suddenly from supposed heart-clot, after a violent altercation with her husband.

Not long after I was called to Mount Holly to see a case which had been correctly diagnosticated by the physician. I removed a petrified seven-months' child. The patient had albuminuria, but did well until the eleventh day, when uræmic convulsions set in, and she died comatose. All these operations were performed before the days of antiseptic surgery, and I have not since seen a case of advanced ectopic gestation.

I believe that Tait is correct in explaining these advanced cases by the rupture of the tube and the escape of the unbroken gestation sac into the fold of the broad ligament. The behavior subsequently is precisely like that of an intra-ligamentary ovarian cyst.

In regard to early diagnosis, I should say that the most common symptom is arrest of menstruation for one or two periods, followed by irregular uterine hemorrhages. It is true that pelvic colic is a common symptom, but not so common as the other. But I no not know that it is necessary to make an absolute diagnosis; given a woman with the exacting symptoms of a suspected extra-uterine fœtation, who has a displacing-tumor on one side of the womb, are we warranted in operating merely to remove the tumor, whatever its nature? Do we not constantly, on less provocation, remove pelvic tumors whose character is determined only by the operation? Instead of an extra-uterine fœtation, we may find pyosalpinx, or an ovarian abscess; but were we not in duty bound to perform the operation, even at the risk of an error in diagnosis?

DR. BARTON C. HIRST.—I was sometime ago called to a case in consultation which presented a clear history of extra-uterine fœtation; cessation of two periods, hemorrhage with the discharge of deciduous membrane, a distinct tumor to one side of the uterus, and the subjective signs of pregnancy, with swelling of the breasts and vomiting. Dr. Hamill and myself urged operation, but the family being dissatisfied, we were discharged. Another physician was called, and Dr. Parrish was consulted. He recommended the use of electricity, and a current was applied with relief of the symptoms, and, I believe, complete cure of the patient. There may be a varicose vein in the broad ligament which, having burst, may present all the signs of extra-uterine fœtation after rupture of the sac. I have had two such cases; in one case I opened the abdomen and found a blood-tumor in the layers of the broad ligament and considerable blood in the peritoneal cavity. From the history and physical signs, I am quite sure that this was not an extra-uterine pregnancy.

I saw, in consultation, a fatal case of this kind after labor not long ago. The labor was a difficult one and ended by craniotomy. There was rupture of a vein in the broad ligament. The bleeding was first between the layers of the broad ligament. This then ruptured into the peritoneal cavity and the woman died. There was no rent in the uterine wall. Such cases might be mistaken for extra-uterine fœtation.

DR. M. PRICE.—In most of these cases, all that we can make out is that there is something which should be removed; but as to a distinct diagnosis of extra-uterine pregnancy being made, I do not believe that it is done one time in ten. Dr. Parvin, in his case, would not have been surprised to have found distention of the tube from any cause. In a case which presented all the symptoms of uterine pregnancy, and where I expected to find this condition, I found a pair of large pus-tubes. I have seen twenty or twenty-five cases of extra-uterine pregnancy, nearly all of them ruptured tubal pregnancies. It does not interest us a particle whether the cases were diagnosed or not. There is trouble present of such a serious character that it does not become us to lose a single moment. Most of these cases come into the coroner's and not the surgeon's hands. Delay in operating is adding ten per cent. to our mortality. It is our duty to operate on the first indication, and if we are mistaken, to thank God for the absence of so serious a condition.

DR. JOSEPH HOFFMAN.—I have twice operated for extra-uterine pregnancy and did not find it. I operated once for something else and found extra-uterine pregnancy. The first case presented the signs of extra-uterine pregnancy to even a more marked degree than that of Dr. Hirst, coming on after a sterility of eight years, a retroverted mass flooding and violent pain. At the operation I found two pus-tubes. She was pregnant and miscarrying and completed it after the operation. The second case was almost on a par with this. In the third case I operated for pus-tube and found extra-uterine pregnancy. The trouble is, that these men who claim positive diagnosis do it from a single case, which, though by no means certain at the time of operation, resolves itself into an absolute diagnosis when they come to publish it. It is the dreams and the nightmare of desire to publish something startling, which make the diagnosis. There are such a variety of conditions, in the pelvis giving rise to these symptoms, that it is impossible to say absolutely what we have. I have gone over Dr. Thomas' list of cases and found that, so far as absolutely correct early diagnosis is concerned, it is worth nothing. In only one case, if my memory serves me, was there a *post mortem.* In three or four in which death occurred there was no *post mortem;* and in the others, where the current was used, the diagnosis in most of them was made after symptoms of rupture.

The treatment by the electric current is advocated by Reeves, who strangely falls into a fallacious argument. He says that if we use electricity in cases where there are symptoms of extra-uterine pregnancy, and these symptoms disappear after the use of the current, why not call them extra-uterine, as we do in other diseases, where diagnosis is made, and a line of treatment adopted with good results, and illustrates the argument by the use of mercury in syphilis. If the effect of electricity was as well established as that of mercury in syphilis, the argument would be more logical. When we feel that rupture has occurred, electricity is a dangerous thing to tamper with. The principal danger is in delay. The longer the growth is allowed to continue, the greater the danger from adhesions, rupture and complications which cannot be foreseen.

Dr. J. PRICE.—There are some interesting facts in connection with the history of this subject. It is curious that a few years ago a man with an experience of one doubtful case should discuss the subject before the American Gynecological Society. It is also curious that the same man, with a single experience with a woman sterile five years, having pelvic pain, irregular menstruation, a delayed period for six days, and recurring attacks of pain, should claim to have killed the fœtus of an extra-uterine pregnancy by the use of electricity for ten *seances* of half an hour's duration each, on consecutive days. Then follows another man with a history of one case, and another in consultation· The man with an experience of one case used electricity, and the case passes into the hands of another, who writes to the first that he is going to operate. The first physician at once writes not to do it, as he has killed the fœtus ; while the operator already holds in his hands a large hydrosalpinx.

Dr. BALDY, in a recent paper in the *New York Medical Record*, lays down certain propositions, and makes the diagnosis easy in many cases. He has forgotten that many of these patients die as if with a ruptured aneurism before the woman suspects that anything is wrong. These cases go to the coroner. In the cases that reach the surgeon the hemorrhage has not been so great.

It is interesting to refer, in this connection, to the cases of Dr. Edis and Dr. Bantock. Dr. Edis found on one side an extra-uterine pregnancy, and on the other a small ovarian cyst. Dr. Bantock found on one side an extra-uterine pregnancy, and a pus-tube on the other. I have here an enormous tubal pregnancy, with rupture at the pavilion, and the abdomen filled with blood. On the other side was a beautiful hydrosalpinx.

One of the prominent cases on record I saw a few hours before the operation. We agreed that there was a doubtful history of extra-uterine pregnancy, but no prominence was placed upon this point. It might have been a small ovarian or dermoid cyst, or a large hydrosalpinx. When the abdomen was opened, every one expressed great surprise at the presence of tubal gestation. The same case has flourished over the world as a case of positive diagnosis. This is the case in which letters were written inviting different men to the operation ; but the letters were written after the operation. This case will not bear close scrutiny. If we look over the transactions of the American

Gynecological Society, we shall find that the men who have had the least experience speak most positively.

Dr. NOBLE.—Monday, a week ago, I removed an extra-uterine pregnancy which was rather unusual in the conditions present. In my case the woman had skipped the period in August. There was nausea and other symptoms of pregnancy. In September, a week before the sickness should have occurred, she began to flow freely; then had cramps and fainted in the street, and afterwards had fainting attacks. I saw her two weeks after the beginning of the flow. At one time, while in the water-closet, she passed what she thought to be a large clot. The opinion of her physician was that she had had a miscarriage.

I found a mass on the left side pushing the womb to the right. I recommended operation, which was not consented to for some days. In the meantime, the hemorrhage continued. The patient was seen by Dr. Kelly, and we agreed that it was almost certainly an extra-uterine pregnancy. At the operation I found the blood in the peritoneal cavity. The blood was almost entirely clotted. The ovum was attached not far from the uterus. The hemorrhage had taken place in the tube, and the clots had been forced out through the fimbriated extremity. On the other side there was a hydrosalpinx. She had also had the rectal tenesmus. She has done well since the operation.

DR. B. F. BAER.—I wish to go on record as one who believes that it is as easy to diagnose extra-uterine pregnancy as to diagnose any other condition within the abdomen (as hydrosalpinx or pyosalpinx) positively. But the man who says he can make such a diagnosis positively is an unsafe man. Dr. Taylor assisted me some five years ago in a case of extra-uterine pregnancy in which the diagnosis was made. The fœtus had advanced to the age of two and a half months, and the symptoms which have been mentioned were present. There was enlargement of the uterus, and I heard the placental bruit. I felt fluctuation in this sac, and was almost certain that I detected ballotment. I, however, operated in an unscientific way, cutting through the vagina. I got the embryo, but the patient died.

DR. M. PRICE.—Did I understand Dr. Baer to say that he heard the placental bruit at two and a half months?

DR. J. M. BALDY.—How did Dr. Baer detect ballotment at this early stage?

DR. BAER.—I have heard this murmur in normal pregnancy at two and a half months. I believe that it is not yet clearly understood whether the murmur is placental or simply a uterine murmur. It is, however, due to pregnancy.

In regard to ballotment, given a sac filled with liquor amnii and an embryo dangling in it, I think that ballotment could be readily detected.

DR. WILLIAM GOODELL.—Dr. Baer is right in regard to early ballotment. This is said to be detected much earlier in extra-uterine fœtation than in natural pregnancy.

DR. J. M. BALDY.—I know there are cases on record, but I doubt the occurrence of ballotment in these. It could not possibly have occurred in such specimens as that presented by Dr. Goodell a few months ago, or those of Dr. Parvin and my own presented to-night. And so it has been with all the cases I have seen. Reference has been made several times to a paper of mine in the New York *Medical Record*, in which I lay down three propositions. The first is, that in a certain proportion of cases of extra-uterine pregnancy in the early stages, the diagnosis is easy and unmistakable. The second, that in a certain (quite large) proportion of cases sufficient symptoms are present to more than warrant a diagnosis of extra-uterine pregnancy, such a pregnancy not being present. The third, that in a certain other proportion of cases the symptoms, until rupture has occurred, are entirely wanting, or are of such a dubious character as to in no wise warrant such a diagnosis. These propositions have been abundantly sustained by the cases reported to-night. Cases have been reported in which the diagnosis was made and the condition found, and there are enough of these cases on record to show that the diagnosis can be made at times. Then we come to the second group of cases, in which the diagnosis is justified by the symptoms, but something else is found. This I sustained by cases of my own, and it has been more than abundantly substantiated by cases reported to-night by Dr. Goodell, Dr. Hirst, Dr. J. Price, Dr. M. Price and Dr. Hoffman. Dr. Johnston, of Washington, has reported a similar case. The third proposition is also sustained by cases reported to-night (in addition to my own) by Dr. Hoffman, Dr. J. Price and others. If there are well substantiated cases in which the diagnosis was not made and extra-uterine pregnancy found, and, again, if we operate for extra-uterine pregnancy, the symptoms justifying such a diagnosis, and something else is found, I cannot see how any sensible man can claim that it is possible to make a positive diagnosis. I agree with Dr. Hoffman that Dr. Thomas' cases are utterly worthless from a diagnostic point of view; nor does Dr. Thomas himself hold that it is always possible. The men who claim that a positive diagnosis can be made have most signally failed. For instance, the case of Mann, of Buffalo, which went into the hands of Wylie, who operated and did not find ectopic gestation; the case which Buckmaster, together with other medical talent, treated with electricity, even to puncturing, I think, and finally pronounced normally pregnant; the case of Kelly, of Philadelphia, the diagnosis of which case has to-night been denied by two eye-witnesses, as well as others.

It is noteworthy that the men who claim the most on this subject have had the least experience. J. M. Baldy,

328 South Seventeenth Street. *Secretary.*

---

The Mississippi Valley Medical Association at its 19th annual meeting held at Evansville, Ind., September 10, 11, 12, 1889, elected the following officers: President, Dr. Jos. M. Matthews, Louisville; 1st Vice-President, Dr. E. R. Early, Ridgway, Pa.; 2d Vice-President, Dr. Y. B. Harvey, Indianapolis, Ind.; Permanent Secretary, Dr. E. S. McKee, Cincinnati; Treasurer, Dr. C. F. McGahan, Chattanooga, Tenn.; Chairman Committee of Arrangements, Dr. J. N. Bloom, Louisville. Next meeting Louisville, September 9, 10, 11, 1890.

